# Protection against Divergent Influenza H1N1 Virus by a Centralized Influenza Hemagglutinin

**DOI:** 10.1371/journal.pone.0018314

**Published:** 2011-03-28

**Authors:** Eric A. Weaver, Adam M. Rubrum, Richard J. Webby, Michael A. Barry

**Affiliations:** 1 Division of Infectious Diseases, Department of Internal Medicine, Mayo Clinic, Rochester, Minnesota, United States of America; 2 Department of Infectious Diseases, St Jude Children's Research Hospital, Memphis, Tennessee, United States of America; 3 Department of Immunology, Mayo Clinic, Rochester, Minnesota, United States of America; 4 Department of Molecular Medicine, Mayo Clinic, Rochester, Minnesota, United States of America; 5 Translational Immunovirology and Biodefense Program, Mayo Clinic, Rochester, Minnesota, United States of America; University of Delhi, India

## Abstract

Influenza poses a persistent worldwide threat to the human population. As evidenced by the 2009 H1N1 pandemic, current vaccine technologies are unable to respond rapidly to this constantly diverging pathogen. We tested the utility of adenovirus (Ad) vaccines expressing centralized consensus influenza antigens. Ad vaccines were produced within 2 months and protected against influenza in mice within 3 days of vaccination. Ad vaccines were able to protect at doses as low as 10^7^ virus particles/kg indicating that approximately 1,000 human doses could be rapidly generated from standard Ad preparations. To generate broadly cross-reactive immune responses, centralized consensus antigens were constructed against H1 influenza and against H1 through H5 influenza. Twenty full-length H1 HA sequences representing the main branches of the H1 HA phylogenetic tree were used to create a synthetic centralized gene, HA1-con. HA1-con minimizes the degree of sequence dissimilarity between the vaccine and existing circulating viruses. The centralized H1 gene, HA1-con, induced stronger immune responses and better protection against mismatched virus challenges as compared to two wildtype H1 genes. HA1-con protected against three genetically diverse lethal influenza challenges. When mice were challenged with 1934 influenza A/PR/8/34, HA1-con protected 100% of mice while vaccine generated from 2009 A/TX/05/09 only protected 40%. Vaccination with 1934 A/PR/8/34 and 2009 A/TX/05/09 protected 60% and 20% against 1947 influenza A/FM/1/47, respectively, whereas 80% of mice vaccinated with HA1-con were protected. Notably, 80% of mice challenged with 2009 swine flu isolate A/California/4/09 were protected by HA1-con vaccination. These data show that HA1-con in Ad has potential as a rapid and universal vaccine for H1N1 influenza viruses.

## Introduction

Annually, 5 – 15% of the world's population is affected by influenza epidemics and have upper respiratory tract infections, 3 to 5 million have severe illness and 250,000 to 500,000 cases result in death [1]. While these normal infections are certainly of concern, natural pandemic influenza outbreaks and intentional releases of pathogenic influenza are of substantially higher concern. A difficult obstacle inherent to current trivalent inactivated vaccine (TIV) production stems from the need to screen and predict which viruses may circulate in the subsequent year. These predictions do not always accurately identify the actual viruses that cause disease that year [2], [3], [4]. In fact, vaccine mismatches occurred in 4 out of 8 (50%) flu seasons in the USA between 1997 and 2005 [5], [6], [7], [8]. In addition to this problem, some of the immunologic effects of TIV reduces its efficacy. For example, the current vaccine only provides short-term immunity [9], the induced immunity is highly strain specific [3], [10], intramuscular delivery does not stimulate high levels of the secretory IgA that is less specific and is more reactive against heterologous viruses [10], [11], [12], [13], [14], and fail to induce cross-protective cellular immunity [15], [16], [17], [18], [19].

In addition to influenza virus based vaccines, alternative approaches have been investigated. These include the use of electroporated DNA expression plasmids, adenovirus vectored vaccines, and universal vaccines based on centralized genes and conserved matrix ectodomains [20], [21], [22], [23], [24]. Centralized genes were first proposed as universal vaccines for HIV [25], [26], [27], [28], [29], [30]. Centralized sequences minimize the degree of dissimilarity between a vaccine strain and contemporary circulating viruses by creating an artificial sequence based on the most common amino acid in each position in an alignment. Other than HIV, centralized genes have also been proposed as universal vaccines for highly pathogenic (H5N1) avian influenza (HPAI) and Chikungunya virus [20], [31], [32].

Recently, swine flu was declared a pandemic. Even with the latest technologies the CDC and the WHO were not able to agree on a vaccine strategy and implement vaccine production in time for the 2009–2010 influenza season [33]. Due to the delay in vaccine availability, the CDC has estimated that between April and November 19^th^ 2009 there were between 34 and 67 million cases of 2009 H1N1, 154,000 and 303,000 2009 H1N1-related hospitalizations, and 7,070 and 13,930 2009 H1N1-related deaths [34]. 2009 H1N1 has also been confirmed in more than 276 pediatric deaths to date. More than double the annual seasonal flu pediatric deaths [34].

To address the issue of pandemic H1N1 influenza virus, we created a centralized H1 HA immunogen. When *in vitro* immune correlates of protection and *in vivo* prophylaxis was compared. We found that the centralized H1 antigen, HA1-con, induced greater and broader immune correlates as compared to two wildtype H1 antigens. We also found that in the case of vaccine mismatch HA1-con could induce more potent protection against lethal wildtype influenza challenges. Based on these data, this strategy may be applicable to the more divergent H1N1 influenza viruses and represents an alternative universal vaccine that could be either stockpiled or included in the current vaccine formulations in the case of H1 seasonal or pandemic mismatch.

## Materials and Methods

### Alignment, Consensus, and Tree Construction

**Figure 1:**
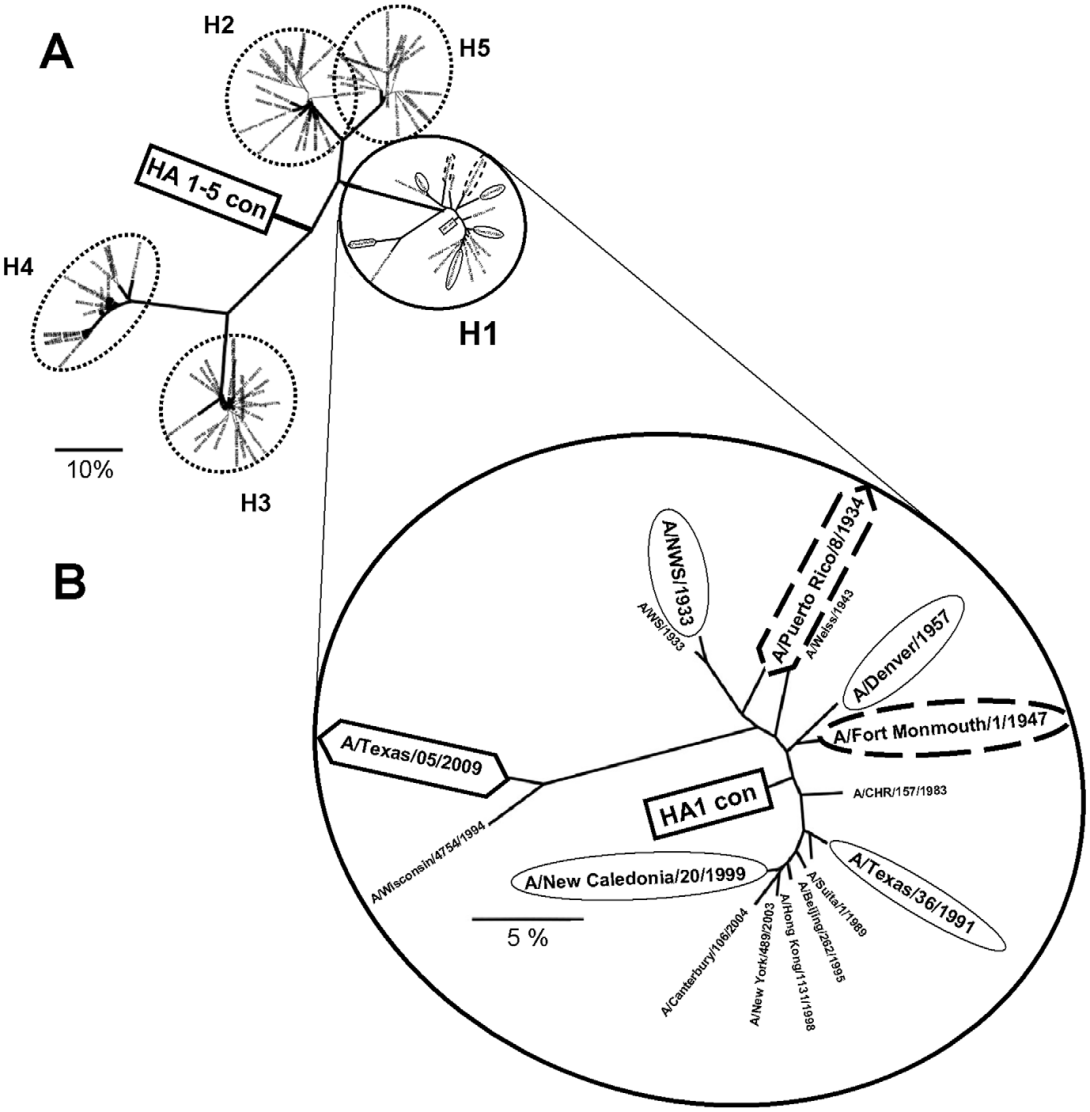
Phylogenetic trees showing the wildtype genetic relationship of wildtype and consensus HA genes. Selected full-length H1 HA sequences were downloaded from NCBI and aligned using Clustal W. The 566 amino acid consensus sequences was determined from this alignment. The HA 1–5 con gene is shown localized to the central region of the multi-subtype phylogenetic tree (A). The HA1-con gene is shown in the phylogenetic H1 subtype tree (B). Unrooted phylogenetic trees were created using PHYLIP version 3.5c. The boxes represent consensus genes used to create the adenovirus vaccines. Diamonds represent wildtype gene used to create adenovirus vaccines. The circles represent viruses or peptides used to evaluate in vitro immune correlates induced by the vaccines. Dashed lines represent viruses used in lethal challenges.

Influenza sequence databases contain a tremendous amount of sequence information. There are over 30,000 HA sequences available through Genbank. Although valuable, not all of this information can be used to create a consensus sequence. During pandemics, epidemics and endemics there is a renewed interest in influenza evolution. At these times multiple nearly identical influenza sequences are entered into the databases from specific institutions diluting out more divergent, but less sequenced isolates. Therefore a consensus sequence created using all of the available sequence information would bias the consensus sequence to that of the most sequenced isolates and those closely genetically related. To reduce this bias, we selected twenty full-length H1 HA sequences from the larger sequence space to represent the main branches of the H1. (accessions: ISDN13422, CY001952, CY003833, CY002392, CY006363, CY007467, CY002624, CY003384, CY003304, CY003696, AF386780, AF386775, AY289928, AF386774, D13574, X17221, U02085, M38312, S62154, and U53162). Novel 2009 H1N1 HA genes were not included in the initial H1 HA consensus design because they had not been identified at the time of analysis. Selected full-length H1 HA sequences were downloaded from Genbank and aligned using Clustal W. All aligned sequences were then inspected manually to correct for apparent mistakes. Positions containing gaps or ambiguously aligned positions were removed from the datasets. The 566 amino acid consensus sequence, named HA1-con, was generated from this alignment by using the most common amino acid at each position. Unrooted phylogenetic trees were created using PHYLIP version 3.5c (Figure 1B). The HA 1–5 consensus gene, HA 1–5 con, was created by the same strategy with the exception that sequences from H1, H2, H3 and H5 were used (Figure 1A) (accessions: D13574, S62154, U02085, NC_002017, CY002624, CY006363, AF386775, AF386780, AY289928, ISDN13422, CY003833, CY002392, CY003384, CY007467, CY003696, AY209963, AY209961, L20409, L11134, D13579, L11126, L11142, L20406, L11125, M54895, AJ289703, V01103, CY006044, CY003064, M55059, CY002056, DQ249261, CY000137, CY002136, AY032978, AB019357, CY002088, X05907, CY002072, CY002904, AJ252131, CY000017, CY003512, CY002744, CY002496, ISDN121986, AB239125, DQ372591, ISDN119678, ISDN117778, ISDN117777, ISDN118371, AJ867074, ISDN110940, AY555150, ISDN40341, AY651334, AY651335, ISDN40278, AY575869, AY575870, ISDN38262, AF102676, AF084279, AF046097, AF084280, and AF084532). N and O linked glycosylation sites for the vaccine genes HA1-con, A/PR/8/34, and A/TX/05/09 were analyzed using NetNGlyc 1.0 and NetOGly 3.1 software analyses.

### Viruses and Vaccines

Influenza virus A/PR/8/34 was obtained from ATCC (VR95). Influenza viruses A/Denver/1/57, A/Fort Monmouth/1/47, A/Texas/36/91, and A/NWS/33 were obtained from the Biodefense and Emerging Infections Research Resources Repository. All of the viruses were passaged one time in SPF embryonated eggs and the chorioallantoic fluid was stored at −80C. The pandemic H1N1 virus used in challenge studies was a mouse-adapted A/California/04/09 virus. This virus was adapted for lethality in mice through serial lung passage as previously described [35]. Stocks of virus were grown in embryonated eggs and the chorioallantoic fluid was stored at -80C).

The influenza virus A/PR/8/34 and A/FM/1/47 stock was titered in BALB/c mice to determine its 50% mouse lethal dose (MLD_50_). The HA sequences for A/PR/8/34, A/TX/05/09 A/HK/213/03 were obtained from NCBI and the Influenza Sequence Database at Los Alamos National Laboratories, respectively. HA1-con, HA 1–5 con, A/PR/8/34, A/TX/05/09, and A/HK/213/03 HA genes were codon-optimized for mammalian expression and synthesized by Genscript. Inc. First generation replication defective (E1/E3 deleted) Ad5 vectors were constructed using the Ad-Easy system in 293A cells as described in [36]. All adenoviruses were purified by CsCl banding and quantitated by OD260. The recombinant Adenoviruses expressing A/PR/8/34, A/TX/05/09, A/HK/213/03, HA1-con and HA1-5 con were named Ad-PR-HA, Ad-TX-HA Ad-HK-HA, Ad-HA1-con, and Ad-HA-1-5-con, respectively. The infectivity of the recombinant adenoviruses was checked using the Adeno-X Rapid titer. There were no statistical differences in infectivity (Figure S1). All virus stocks were found to contain ≤0.1% replication competent adenovirus.

### Animals

Female BALB/c mice (6–8 weeks old) were purchased from Charles River Laboratories (Wilmington, Massachusetts, USA) and housed in the Mayo Clinic or St Jude Children's Research Hospital Animal Facility under the Association for Assessment and Accreditation of Laboratory Animal Care (AALAC) guidelines with animal use protocols approved by the corresponding the Mayo Clinic Institutional Animal Care and Use Committee (IACUC protocol No. A110). All animal experiments were carried out according to the provisions of the Animal Welfare Act, PHS Animal Welfare Policy, the principles of the NIH Guide for the Care and Use of Laboratory Animals, and the policies and procedures of Mayo Clinic and St Jude Children's Research Hospital.

Mice were anesthetized i.p. with ketamine (140 mg/kg)/kylazine (5.55 mg/kg) and were immunized intramuscularly (i.m.) with various doses of Adenovirus in a volume of 50 ul. Twenty-five µl was injected into each mouse quadriceps. Three weeks post-immunization the mice were challenged intranasally with mouse-adapted influenza virus A/PR/8/34, A/FM/1/47, or A/California/04/09.

### Cellular Immunity: Enzyme-Linked Spot (ELISPOT) Assay

Mice were immunized i.m. with 10^10^ virus particles (vp) of adenovirus expressing HA genes from PR, TX or H1 consensus. Three weeks post-immunization the mice were sacrificed and spleens were harvested. ELSIPOT assays were performed as previously described [37]. Overall cellular immune responses were measured using three pools of a peptide array, Influenza Virus A/New Caledonia/20/99 (H1N1) Hemagglutinin Protein. The peptides consisted of 94 16- to 17-mers, with 11 or 12 amino acid overlaps and were obtained from the Biodefense and Emerging Infections Research Resources Repository (Catalog No. NR-2602). Then using a matrix of peptide pools and individual peptides the immuno-stimulatory epitopes were mapped. Concanavalin A (5 µg/ml) was used as a positive control while splenocytes from DPBS immunized animals as well as media only wells were used as negative controls. Responses were considered as positive if the number of the spots was four-fold higher than that of the negative control and at least 50 SFC/10^6^ cells.

### Humoral Immunity: Hemaglutination Inhibition (HI) Assay

Mice were immunized i.m. with 10^10^ vp of adenovirus expressing HA genes from PR, TX or H1 consensus. Three weeks post-immunization the mice were bled by cardiac puncture and sacrificed. Serum was collected using Becton Dickinson microtainer tubes with serum separator. Starting at a dilution of 1∶5 sera were diluted two-fold in 50 µl of DPBS in a 96-well, nonsterile, nontissue culture–treated, round bottom microtiter plate. Four HAU of influenza virus in 50 ul was added to the diluted sera and incubated at room temperature (RT) for 1 hr. After incubation, 50 µl of a 1% chicken RBC solution was added and incubated at RT for 1 hr. The HI titer was determined to be the highest serum dilution to inhibit hemagglutination.

### Influenza Challenge

Mice were immunized with various doses of the Ad-vectored vaccines. Three weeks after immunization the mice were anesthetized i.p. with ketamine (140 mg/kg)/xylazine (5.55 mg/kg). The mice were weighed for baseline measurements. The mice were challenged intranasally with 100LD_50_ of influenza A/PR/8/34 or A/FM/1/47 virus or 70LD_50_ of A/California/04/09 virus. The mice were placed on their backs and 10 µl of A/PR/8/34 or A/FM/1/47 virus was pipetted into each nare for a total volume of 20 µl. For infection with A/California/04/09, 15 µl was delivered to each nare for a total of 30 µl. The mice were then weighed and monitored daily for signs of disease. Mice were humanely euthanized if their body weight dropped to 75% of baseline weights.

## Results

### Production of Centralized Consensus HA Immunogens

A comparison of select H1 hemagglutinin (HA) protein sequences from 1933 through 2009 generated a phylogenetic tree with ∼21.0% of sequence divergence across the branches (Figure 1B). Due to high levels of genetic diversity, selecting a single wildtype HA protein as a universal vaccine is not thought to be feasible. Rather than select one wildtype gene as a vaccine, we generated a centralized gene that mimics an ancestor of influenza infections during the past 76 years. The rationale for this approach is to produce an immunogen that is centrally located with respect to all other variants. Such a protein then practically has lower sequence divergence with all of the variants than any two randomly selected genes.

Phylogenetic analysis shows that the synthetic centralized HA1-con protein localizes to the central region of the H1 tree (Figure 1B). When genetic distances were calculated using ClustalW, the HA1-con was found to be half the genetic distance to the wildtype influenza strains used in this study (Table 1). For example, influenza A/FM/1/47 was used as a challenge strain in subsequent studies using three homologous vaccines, HA1-con, A/TX/05/09 and A/PR/8/34. When the genetic distances from the mismatched vaccines to the challenge strain were calculated, the HA1-con was closest genetically with 4.8% divergence. A/TX/05/09 and A/PR/8/34 were found to be 19.6% and 9.7% divergent, respectively (Table 1). When vaccinated mice were challenged with A/PR/8/34 the mismatched vaccines HA1-con and A/TX/05/09 were 8.5% and 18.6% divergent, respectively. Again, HA1-con is closest genetically when the vaccine and challenge strain are mismatched. Although HA1-con was not half the genetic distance to the A/CA/04/09 flu challenge strain as compared to the mismatched A/PR/8/34, it was slightly closer (Table 1).

**Table 1:** Clustal Distance Matrix.

	A/FM/1/47	A/NWS/33	A/TX/36/91	A/Denver/1/57	A/New Caledonia/20/99	A/PR/8/34	A/CA/04/09
A/PR/8/34	0.097	0.078	0.122	0.115	0.126	0.000	0.186
A/TX/05/09	0.196	0.182	0.208	0.202	0.202	0.186	0.005
A/HK/213/03	0.374	0.362	0.369	0.317	0.368	0.355	0.371
**HA1-con**	**0.048**	**0.083**	**0.055**	**0.078**	**0.059**	**0.085**	**0.173**
**HA 1–5 con**	**0.317**	**0.313**	**0.313**	**0.366**	**0.306**	**0.315**	**0.318**

Distance matrix calculated using ClustalW with no exclusions of positions with gaps and no correction for multiple substitutions.

**Figure 2:**
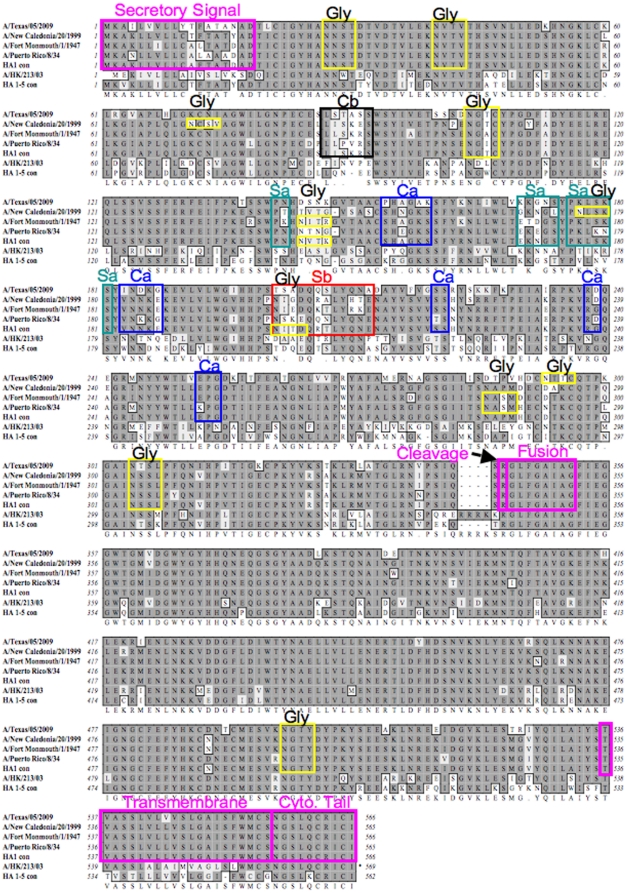
Alignment of the centralized influenza vaccine genes, the wildtype virus genes and the A/New Caledonia/20/99 HA proteins showing the location and sequence of the major antigenic sites Sa, Sb, Ca and Cb. The conserved functional elements consisting of the secretory signal, cleavage, fusion, transmembrane and cytoplasmic tail are indicated and boxed. N-linked glycosylation sites (Gly) are shown boxed in yellow.

Alignment of HA amino acid sequences showed that the HA1-con protein conserved functional elements that include cleavage, fusion, transmembrane and cytoplasmic domains (Figure 2). Comparison of predicted N and O linked glycosylation sites for HA1-con to HAs from A/PR/8/34 and A/TX/05/09 indicated that all of the N-glycosylation sites for A/TX/05/09 and A/PR/8/34 were present in HA1-con (Figure 2). In addition, there were two additional potential N-glycosylation sites in the HA1-con at positions 144 and 201. The predicted N-glycosylation at position 144 was also found in several other isolates such as A/FM/1/47 and A/CHR/157/83. The predicted N-glycosylation at position 201 in HA1-con was not present in the subset of HA proteins analyzed, but represents a conserved asparagine sequence from the 20 HA proteins used for construction. No O-glycosylation was predicted in HA1-con, A/PR/8/34, or A/TX/05/09 (Figure 2).

A broader HA consensus protein (HA 1–5 con) was engineered as a centralized immunogen spanning H1, H2, H3, H4, and H5 influenza viruses (Figure 1A). Although H4 influenza infections have yet to be discovered in humans, it was included in the tree construction to illustrate that the HA 1–5 con gene localizes centrally to this subtype as well. Influenza has a tremendous amount of evolutionary plasticity in its HA sequences as H3 and H5 proteins, for example, are as much as 60% divergent (Figure 1A). A blast search of the Genbank viral sequences database of the HA 1–5 con protein sequence revealed the closest influenza isolates had only 69% identity. All of the closest isolates were H1N1 viruses and included A/SouthCarolina/1/1918 (H1N1), A/swine/Jamesburg/1942 (H1N1), A/swine/Iowa/1945 (H1N1), and A/duck/Italy/281904/2006 (H1N1). Table 1 shows that the genetic distance between HA 1–5 con and all of selected H1 isolates is equally distant from them as the H5 influenza virus A/Hong Kong/213/2003 (A/HK/213/03). Based on conventional wisdom, the amino acid divergence of this synthetic HA 1–5 con gene would not be predicted to induce protection against H1 influenzas. Based on this, a codon-optimized gene for HA 1–5-con was constructed and used to generate the vaccine Ad HA 1–5-con. This vector was included as a divergent immunogen to act as a likely negative control for the HA1-con vaccine, but also as a long-shot cross-reactive vaccine against many influenza viruses.

### Intrasubtype Protection at High Doses of Vaccine

**Figure 3:**
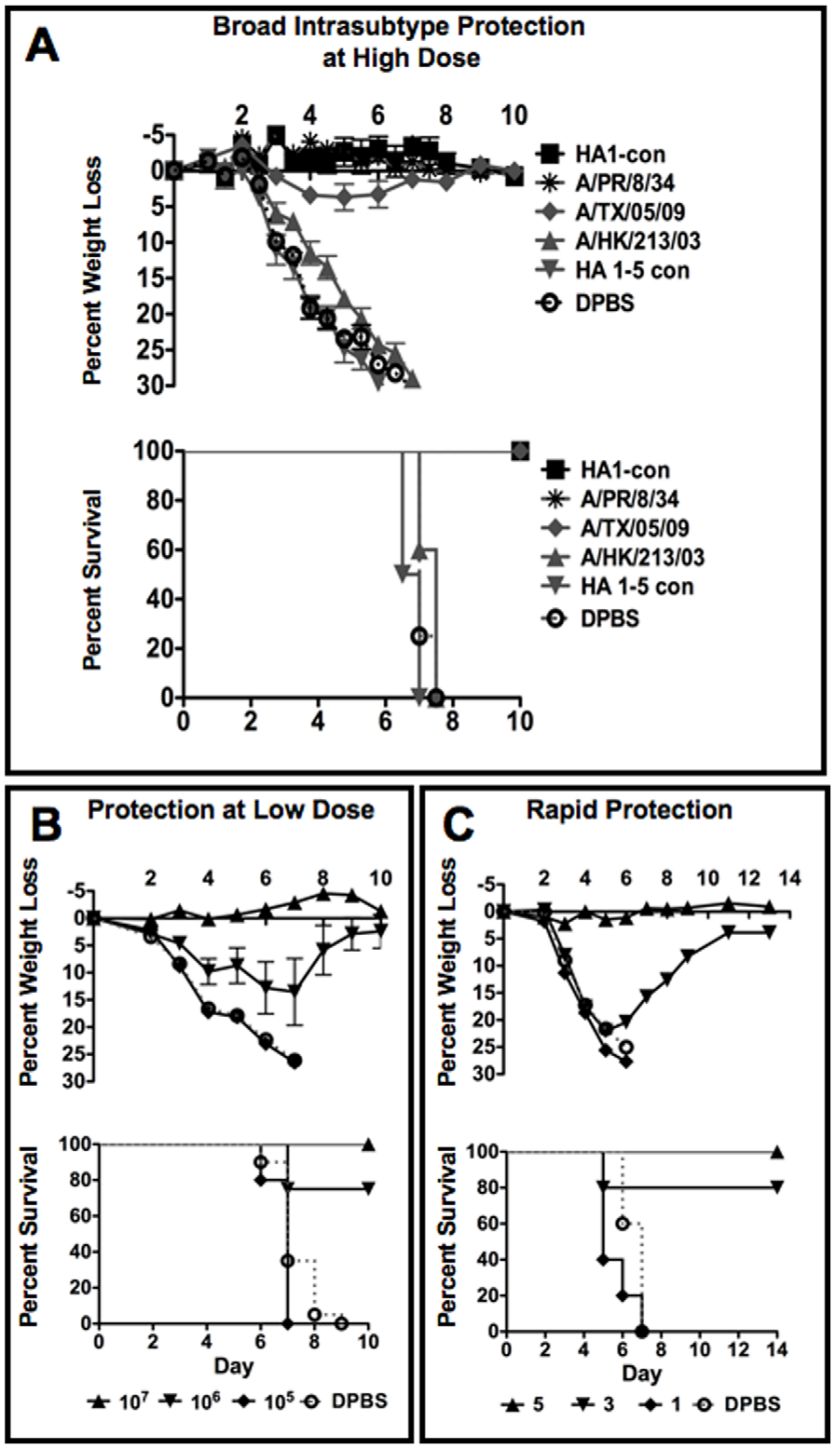
Adenovirus as a platform for Influenza vaccines. Prophylactic responses in mice immunized with consensus and wildtype adenoviral vectors were determined by immunizing mice with 10^10^ Ad vaccine viral particles. Three weeks after immunization the mice were challenged intranasally with 100 MLD_50_ of influenza A/PR/8/34 virus (A). In order to determine how much ad vaccine would be required to induce prophylaxis, mice were immunized intramuscularly with 10-fold dilutions of adenovirus expressing the A/PR/8/34 HA and challenged with 100 MLD_50_ of influenza A/PR/8/34 virus 3 weeks later (B). In order to determine the length of time to induce prophylaxis and the duration of prophylactic immune responses using Ad vaccines, mice were immunized intramuscularly with 10^10^ vp of Ad expressing A/PR/8/34 HA. The mice were challenged intranasally with 100 MLD_50_ of influenza A/PR/8/34 virus 1, 3, 5 and 200 days post immunization (C). Weight loss and survival were monitored daily. Mice that lost more than 25% of their baseline weight were humanely sacrificed.

Mice were immunized with 10^10^ virus particles (vp) of all of the Ad influenza vaccines. Three weeks later, the immunized mice received a lethal challenge with one hundred times the mean lethal dose (100 LD_50_) of influenza A/PR/8/34 virus (Figure 3A). Only HA1-con and the cognate immunogen A/PR/8/34 HA were able to protect mice from disease, weight loss, and death. A/TX/05/09 HA was able to protect the mice from death. However, there were still signs of disease and weight loss using a large dose of vaccine. As predicted the other vaccines were unable to protect against disease and death.

### Protection Against Homologous Challenge at Low Dose

Direct comparison of gene-based vaccines in non-human primates has demonstrated that Ad vaccines generate more robust immune responses and protection than plasmid or vaccinia vaccines [38]. Given our goal to develop a gene-based vaccine for influenza, we tested this vaccine platform by inserting the codon-optimized cDNA for HA from H1 influenza A/Puerto Rico/8/1934 (A/PR/8/34) into a E1-deleted replication-defective Ad5 vector (Ad-PR-HA). To titrate the efficacy of the vaccine, groups of 5 female BALB/c mice were immunized with a range of doses of Ad-PR-HA by the intramuscular (i.m.) route and were challenged 3 weeks later with 100 LD_50_ of influenza A/PR/8/34 (Figure 3B). A vaccine dose of 10^7^ vp was capable of completely protecting mice from death and disease. An even lower dose of 10^6^ vp demonstrated reduced weight loss and increased survival against this stringent lethal challenge.

### Rapid Protection Against Stringent Lethal Challenge

Ad vectors produce rapid and high levels of protein production *in vivo* [39]. To assess the kinetics of Ad vaccine protection, groups of 5 BALB/c mice were vaccinated with 10^10^ vp of Ad-PR-HA by the i.m. route and then challenged with 100 LD_50_ of A/PR/8/34 influenza 1, 3, or 5 days after vaccination (Figure 3C). Under these conditions, mice were fully protected from this challenge within 5 days of vaccination. In addition, mice vaccinated 3 days before challenge showed clinical signs of disease and weight loss, but most survived this stringent challenge. These data suggest that Ad vaccines may generate rapid protection against influenza.

### Cross-reactive Cellular Immune Responses

**Figure 4:**
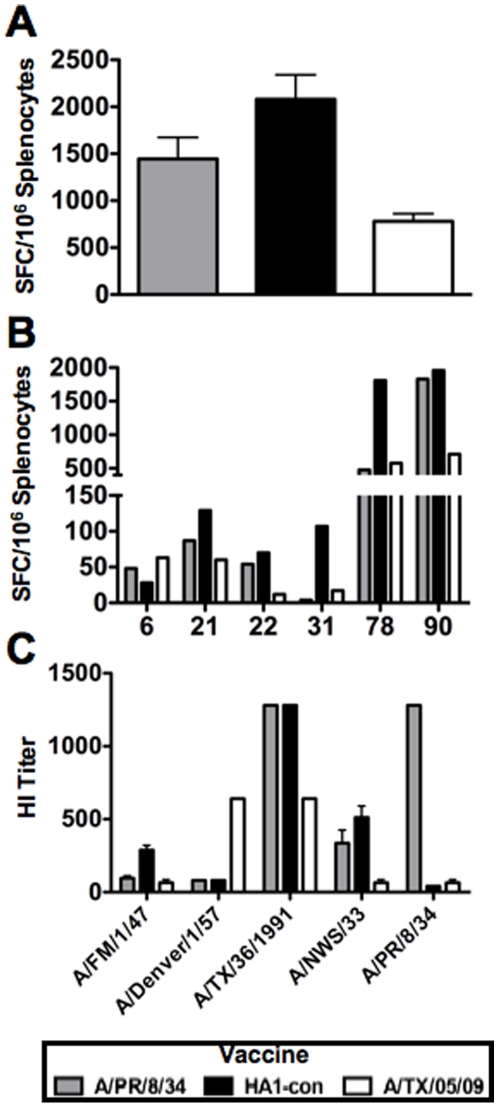
Immune correlates of Protection. Mice were immunized with 10^10^ Ad vaccine viral particles. Three weeks post-immunization the mice were bled and spleens were removed for anti-influenza humoral and cellular responses. Splenocytes were stimulated with peptide pools representing full-length A/New Caledonia/20/99 HA protein in order to determine the overall magnitude of wildtype cellular immune responses (A). Individual peptides were used to identify specific epitopes recognized by splenocytes from immunized mice. The peptide numbers that resulted in positive cellular responses are shown (B). Hemagglutination inhibition (HI) titers induced by the vaccine genes against wildtype viral isolates (C). Groups of 5 mice were used and error bars represent standard error.

Three weeks after i.m. immunization with 10^10^ vp of recombinant adenovirus, the mice were sacrificed and splenocytes were harvested for ELISPOT assays (Figure 4A and B). Splenocytes were stimulated with overlapping peptides from the heterologous virus A/New Caledonia/20/99 (Figure S2). Both A/PR/8/34 and HA1-con immunized mice induced significantly greater cellular immune responses than A/TX/05/09 immunized mice with p values of 0.026 and 0.001, respectively (Figure 4A). Individual immunostimulatory peptides were identified using overlapping peptide pools and individual peptides (Figure 4B). Greater than 50 interferon-γ spot-forming cells (SFC) were considered significant. Epitope mapping revealed that five epitopes (6, 21/22, 31, 78 and 90) were recognized by splenocytes from the immunized mice (Figure 4B). HA1-con generated greater T cell responses against all peptides than either A/PR/8/34 or A/TX/05/09 with the only exception being against peptide 6. T cells from HA1-con immunized mice reacted against two dominant epitopes 78 and 90 while A/PR/8/34 only induced one dominant T cell response against epitope 90. In addition HA1-con immunized mice induced T cell responses against epitope 31 while A/PR/8/34 and A/TX/05/09 immunized mice did not. Interestingly, almost all of these epitopes were entirely conserved in all of the genes (Figure S2). There were two amino acid substitutions in epitope 31 that were not in HA1-con. HA1-con immunized mice induce much stronger T cell immune responses against epitope 78, however there were no sequence differences between the HA1-con and A/PR/8/34 and A/TX/05/09 at that site.

### Cross-reactive Humoral Immune Responses

Groups of 10 BALB/c were immunized with 10^10^ vp of Ad vaccine. Three weeks after immunization, sera from the mice were tested for hemagglutination inhibition (HI) antibody responses against a series of wildtype H1 influenza viruses (Figure 4C). HA1-con induced HI titers that were equal or greater than those induced by A/PR/8/34 or A/TX/05/09 against isolates A/FM/1/47, A/TX/36/1991 and A/NWS/33. HA1-con immunized mice generated significantly higher HI titers against A/FM/1/47 influenza than A/PR/8/34 (p = <0.001). HA1-con induced significantly greater HI titers against A/FM/1/47 and A/NWS/33 viruses than A/TX/05/09 (p = <0.001). However, A/TX/05/09 did induce significantly higher HI titers against A/Denver/1/57 than both Ad-PR-HA and Ad-HA1-con (p = <0.001). Values were log transformed for statistical analyses.

### Protection Against Lethal A/PR/8/34 Influenza

**Figure 5:**
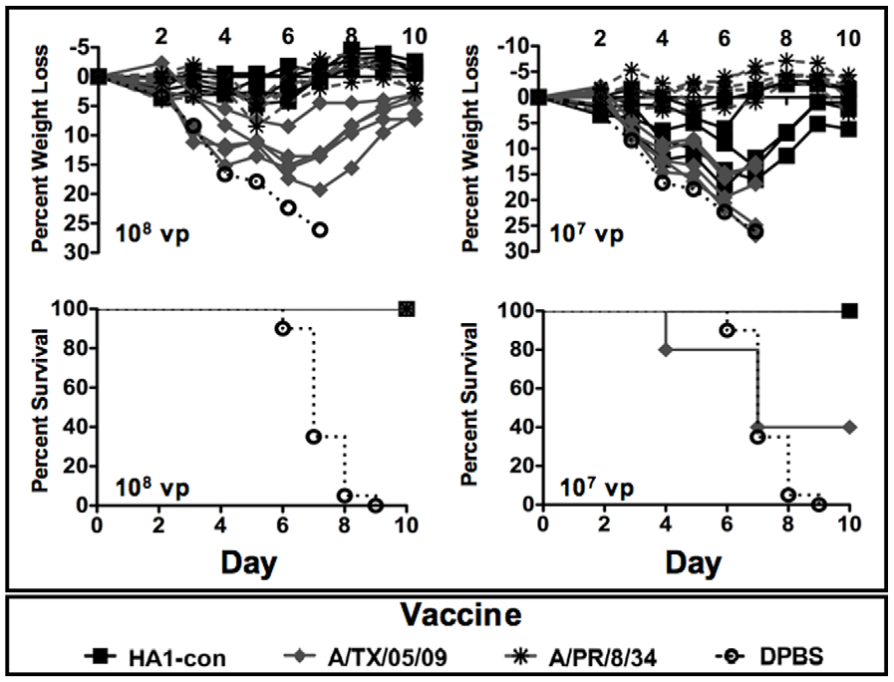
Protection against A/PR/8/34 influenza virus infection. Mice were immunized intramuscularly with various doses of A/PR/8/34, Ad-HA1-con, and A/TX/05/09 HA expressing virus. Three weeks after immunization the mice were challenged intranasally with 100 LD_50_ of influenza virus A/PR/8/34. Individual mouse weights for the vaccinated mice and the mean and standard error of the control DPBS immunized mice are shown. Mice exhibiting profound signs of disease and less than 75% of baseline weights were humanely sacrificed.

As expected the homologous A/PR/8/34 vaccine was able to induce protective responses using the lowest dose of vaccine as compared to the mismatched vaccines, HA1-con and A/TX.05/09 (Figure 5). A/PR/8/34 immunized mice did not show any signs of disease or death with doses as low as 10^7^ vp (Figure 5). Immunization with 10^6^ vp did not prevent disease, but did provide protection against disease in 75% of mice. Doses lower than 10^6^ vp resulted in disease and death (Figure S3). In regard to the vaccine mismatches, the HA1-con was able to protect against disease at a dose of 10^8^ vp while A/TX/05/09 vaccinated mice exhibited signs of disease and weight loss. Both HA1-con and A/TX/05/09 were able to protect against death at a dose of 10^8^ vp. Mice vaccinated with HA1-con at a dose of 10^7^ vp did show signs of disease and weight loss. However, these mice recovered and survived the challenge whereas 60% of mice vaccinated with A/TX/05/09 at the same dose did not survive (Fig 5) (p = 0.05). Doses lower than 10^7^ vp resulted in disease and death (Figure S3).

### Protection Against Lethal A/FM/1/47 Influenza

**Figure 6:**
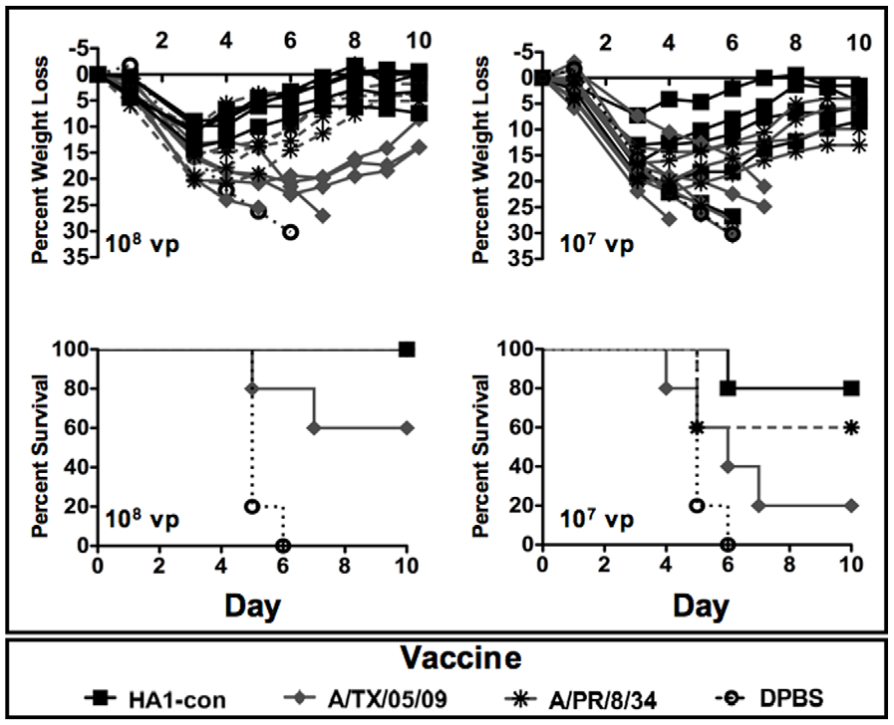
Protection against A/FM/1/47 influenza virus infection. Mice were immunized intramuscularly with various doses of A/PR/8/34, Ad-HA1-con, and A/TX/05/09 HA expressing virus. Three weeks after immunization the mice were challenged intranasally with 100 LD_50_ of influenza virus A/FM/1/47. Individual mouse weights for the vaccinated mice and the mean and standard error of the control DPBS immunized mice are shown. Mice exhibiting profound signs of disease and less than 75% of baseline weights were humanely sacrificed.

In contrast to the homologous A/PR/8/34 virus challenge, A/PR/8/34 HA was not able to induce protection from disease and death from lethal A/FM/1/47 at a dose of 10^7^ vp (Figure 6). In fact, doses up to 10^9^ vp of Ad-PR-HA were unable to prevent disease and weight loss (Figure S4). The mismatched A/TX/05/09 vaccine was even less effective at inducing protective responses against the influenza A/FM/1/47 virus lethal challenge. Doses of 10^9^ vp of A/TX/05/09 were unable to protect against death. However, Ad-HA1-con was able to induce protection against weight loss at a dose of 10^9^ vp (Figure S4). Vaccination with a dose of 10^8^ vp of HA1-con provided the best protection against weight loss and death (Figure 6). Both HA1-con and A/PR/8/34 protected against death while A/TX/05/09 did not resulting in only 60% survival. A lower dose, 10^7^ vp, resulted in weight loss and death in all groups, however, the HA1-con group were best protected resulting in less average weight loss and the best survival (Figure 6). Mice immunized with 10^7^ vp of HA1-con resulted in 80% protection against death whereas only 20% of A/TX.05/09 immunized mice were protected from death (p = 0.06). Doses lower than 10^7^ vp resulted in significant weight loss and death in all groups. However, the only mouse to survive after immunization with 10^6^ vp was in the HA1-con group (Figure S4).

### Protection Against Lethal A/California/04/09

**Figure 7:**
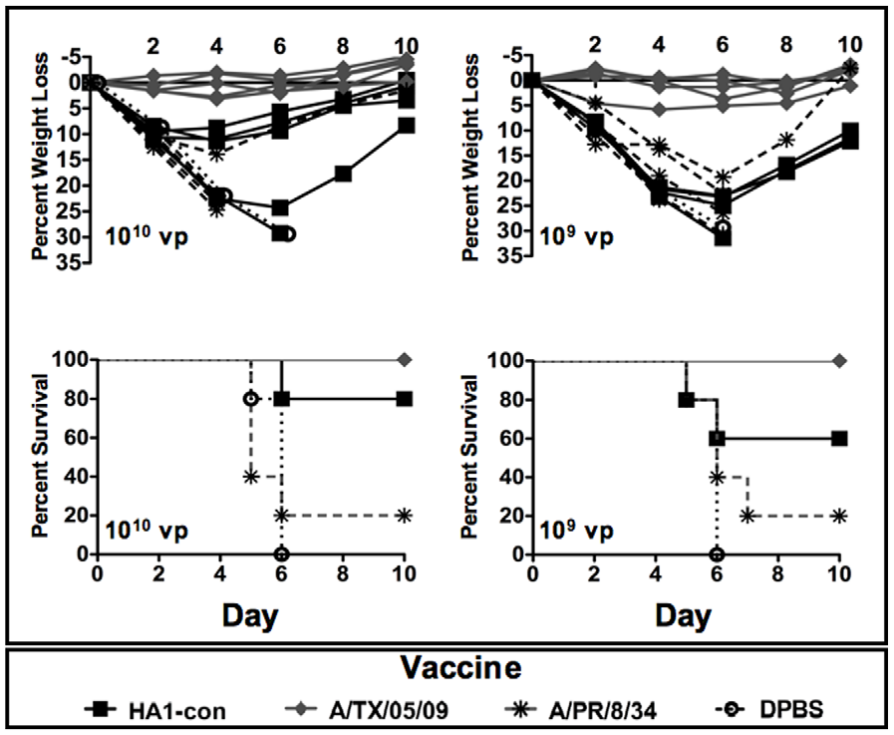
Protection against A/CA/04/09 influenza virus infection. Mice were immunized intramuscularly with various doses of A/PR/8/34, Ad-HA1-con, and A/TX/05/09 HA expressing virus. Three weeks after immunization the mice were challenged intranasally with 70 LD_50_ of influenza virus of A/CA/04/09. Individual mouse weights for the vaccinated mice and the mean and standard error of the control DPBS immunized mice are shown. Mice exhibiting profound signs of disease and less than 75% of baseline weights were humanely sacrificed.

As predicted the A/TX/05/09 vaccine provided the best levels of protection against a lethal challenge with A/CA/04/09 (Figure 7). Similarly, A/TX/05/09 protected against death at a dose of 10^7^ vp and provided some protection at a dose of 10^6^ vp as did A/PR/8/34 against a homologous lethal challenge. A/TX/05/09 vaccine provided complete protection against weight loss and disease at a dose of 10^8^ vp (Figure S5). When the vaccines were mismatched, the centralized HA1-con proved to be better at inducing protective immunity as compared to A/PR/8/34 (Figure 7). At higher doses HA1-con vaccinated mice showed less average weight loss and were better protected against death than A/PR/8/34 vaccinated mice resulting in 80% and 20% survival, respectively. While there were no significant differences in survival between mice immunized with 10^10^ vp of either HA1-con or A/TX/05/09, there were significant differences between A/TX/05/09 and A/PR/8/34 survival rates (p = 0.01). At a dose of 10^9^ vp 60% of HA1-con vaccinated mice survived while only 20% of A/PR/8/34 vaccinated mice survived (Figure 7). There were no statistical differences in survival between mice immunized with 10^9^ vp of A/TX/05/09 or HA1-con. However, A/PR/8/34 ad A/TX/05/09 immunized mice did have significantly different survival rates (p = 0.01). All doses lower than 10^9^ vp of mismatched vaccine resulted in death (Figure S5).

## Discussion

We tested a centralized gene for use as a universal vaccine against H1N1 influenza viruses that might be relevant to seasonal and pandemic influenza. Phylogenetic analysis of HA1-con showed that it did localize to the central region of the phylogenetic tree and was, for the most part, genetically equidistant to the majority of wildtype isolates. *In vitro* characterization showed that an adenoviral vectored HA1-con gene could induce immune correlates in mice equal to or greater than that induced by wildtype genes. Results from the ELISPOT assay indicates that HA1-con induces stronger and broader T cell immune responses in splenocytes. While these cellular immune responses are the primary means by which an influenza viral infection is cleared they are not essential to prevent infection. Humoral immune responses as measured by the HI assay indicate that HA1-con could induce protective responses against all four wildtype isolates tested, providing that the standard measure of protection is an HI titer of 40 or higher [40]. The immune correlates presented here are limited. Intranasal IgA and lung cellular immune responses are also critical immune correlates of protection. However, since we planned to evaluate the vaccine-induced immune responses using a virus challenge advanced immune assays were not necessary. Also, the dose used for this initial evaluation was considerable and would not be practical if scaled up for use in humans.

The ultimate proof of a better vaccine lies in the data from the challenge studies. Not only did the HA1-con centralized gene induce protective responses against all three H1N1 lethal challenges, it was a superior vaccine as compared to all other mismatch vaccines. Although HA1-con did provide better protection against A/California/04/09 than the mismatched A/PR/8/34, these protective responses were at high doses of vaccine. However, these studies were done using high doses of pathogenic influenza virus. The use of 100 LD_50_ is not representative of any epidemic or pandemic ever recorded. Therefore, protective responses against infectious, but not lethal influenza virus may be achieved with even lower doses of adenovirus vectored vaccines. It was surprising to find that matched Ad vectored vaccines provided protection against a very stringent lethal challenge at very low doses. Doses as low as 10^6^ vp of Ad (4×10^7^ vp/kg) reduced weight loss and increased survival against stringent lethal challenge. Extrapolation of this vaccine dose to a 70 kg human suggests that Ad vaccine production from a simple cell factory of 10^9^ cells would produce as many as 1,000 estimated human doses of influenza vaccine. Given that it requires up to 3 eggs to produce one human dose of current trivalent vaccine, this suggests that Ad may be a viable platform for human vaccines. This combined with the observed rapid protection within 5 days of immunization make Ad an attractive influenza vaccine platform to combat seasonal and epidemic spread of influenza.

Another interesting aspect of this study was the creation and testing of a multi-subtype centralized gene, HA 1-5 con. One of the attractive qualities of a centralized immunogen is that conserved or “shared” regions or epitopes would be incorporated into the gene. However, it could be that in the case of high levels of divergence (i.e. inter-subtype) these conserved epitopes would be lost. From a viral evolution standpoint it is interesting that there do not appear to be intermediate HA sequences found in nature that are similar to the centralized HA 1-5 con. However, within the subtypes there are naturally occurring sequences that are similar to HA1-con. In addition to the absence of viral intermediates similar to the HA 1-5 con gene, it is at least ∼30% divergent from all reported wildtype isolates. Given H5 and H1 genes are similarly divergent and that H5 genes do not protect against H1 wildtype virus, it is very possible that HA 1-5 con may simply act as a control HA gene for further studies.

Although the centralized influenza genes proposed in this study are for use as human vaccines, the same concepts and perhaps the same genes may be used to vaccinate reservoir animals against influenza infections. Vaccination of reservoir animals at the source of virus evolution could intervene at this primary step and could result in the elimination of potential future influenza outbreaks that result in pandemics. To this end, our Ad virus vector platform may also be applicable in this approach and may prove to be more potent and cost-effective than traditional vaccines.

While previous studies have reported on the efficacy of consensus H5 genes conferring protection against wildtype influenza virus infection, this is the first reported study that has applied this concept to the more diverse H1 subtype. Here we show that HA1-con is a superior vaccine in the case of mismatch only and produced equal or greater cross-protective immune correlates against wildtype viruses as compared to wildtype HA gene vaccines. However, in the case of a matched vaccine the homologous vaccine gene is superior to HA1-con. For example, Ad-PR induces superior levels of protection against A/PR/8/34 virus as compared to the centralized HA1-con.These data support the concept of using consensus genes as influenza vaccines either as single vaccines or as a “platform” gene to provide broad immunologic cross-reactivity for combination with other single isolate influenza genes.

## Supporting Information

Figure S1 **In order to determine the quality of the adenoviral vectors being used the infectivity of the preps were analyzed using the AdenoX rapid titer kit.** 293 cells were infected with each of the viral preps incubated overnight and stained for hexon expression. There were no significant differences in the Adenoviral vaccine preps.
(TIF)

Figure S2 **Alignment of the consensus influenza vaccine genes, the wildtype virus genes and the A/New Caledonia/20/99 HA proteins.** Numbers represent the epitopes identified in Figure 4. Boxes represent the individual peptides that were recognized by immunized mice splenocytes. The green box represents a unique epitope recognized only by Ad-HA1-con immunized splenocytes and the red box represents the conserved immunodominant CTL epitope. Groups of 5 mice were used and error bars represent standard error.
(TIF)

Figure S3 **Dose-dependent prophylactic responses against a lethal A/PR/8/34 influenza virus challenge.** Mice were immunized intramuscularly with various doses of A/PR/8/34, Ad-HA1-con, and A/TX/05/09 HA expressing virus. Three weeks after immunization the mice were challenged intranasally with 100 LD_50_ of influenza virus A/PR/8/34. Weight loss and death in mice immunized 10^6^ vp are shown in A and B, respectively. Weight loss and death in mice immunized 10^5^ vp are shown in C and D, respectively. The mean and standard error of the control DPBS immunized mice are shown. Mice exhibiting profound signs of disease and less than 75% of baseline weights were humanely sacrificed.
(TIF)

Figure S4 **Dose-dependent prophylactic responses against a lethal A/FM/1/47 influenza virus challenge.** Mice were immunized intramuscularly with various doses of A/PR/8/34, Ad-HA1-con, and A/TX/05/09 HA expressing virus. Three weeks after immunization the mice were challenged intranasally with 100 LD_50_ of influenza virus A/FM/1/47. Weight loss and death in mice immunized 10 vp are shown in A and B, respectively. Weight loss and death in mice immunized 10^6^ vp are shown in C and D, respectively. Weight loss and death in mice immunized 10^5^ vp are shown in E and F, respectively. The mean and standard error of the control DPBS immunized mice are shown. Mice exhibiting profound signs of disease and less than 75% of baseline weights were humanely sacrificed.
(TIF)

Figure S5 **Dose-dependent prophylactic responses against disease after a lethal A/CA/04/09 Swine flu influenza virus challenge.** Mice were immunized intramuscularly with various doses of A/PR/8/34, Ad-HA1-con, and A/TX/05/09 HA expressing virus. Three weeks after immunization the mice were challenged intranasally with 100 LD_50_ of influenza virus 2009 Swine Flu. Weight loss and death in mice immunized 10^8^ vp are shown in A and B, respectively. Weight loss and death in mice immunized 10^7^ vp are shown in C and D, respectively. Weight loss and death in mice immunized 10^6^ vp are shown in E and F, respectively. Weight loss and death in mice immunized 10^5^ vp are shown in G and H, respectively. The mean and standard error of the control DPBS immunized mice are shown. Mice exhibiting profound signs of disease and less than 75% of baseline weights were humanely sacrificed.
(TIF)
