# Supplementary figures and images for: Protection against Divergent Influenza H1N1 Virus by a Centralized Influenza Hemagglutinin
Source: PLoS One. 2011 Mar 28;6(3):e18314. doi: 10.1371/journal.pone.0018314 (PMC3065472; doi:10.1371/journal.pone.0018314)

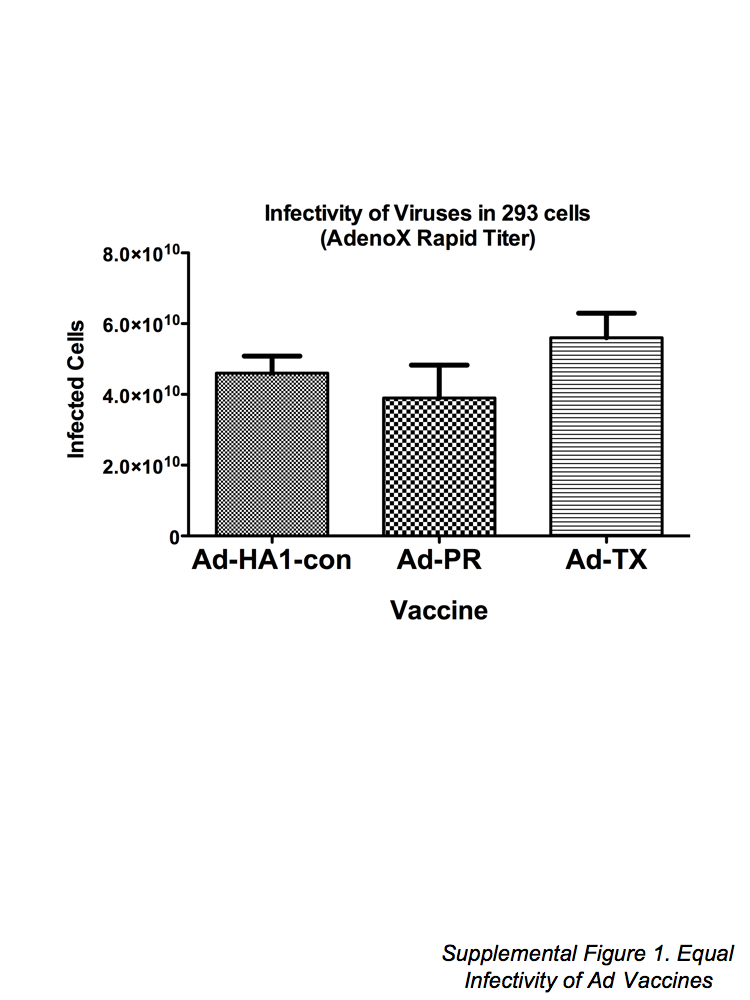

Supplement: Figure S1 — In order to determine the quality of the adenoviral vectors being used the infectivity of the preps were analyzed using the AdenoX rapid titer kit. 293 cells were infected with each of the viral preps incubated overnight and stained for hexon expression. There were no significant differences in the Adenoviral vaccine preps. (TIF) [file pone.0018314.s001.tif]

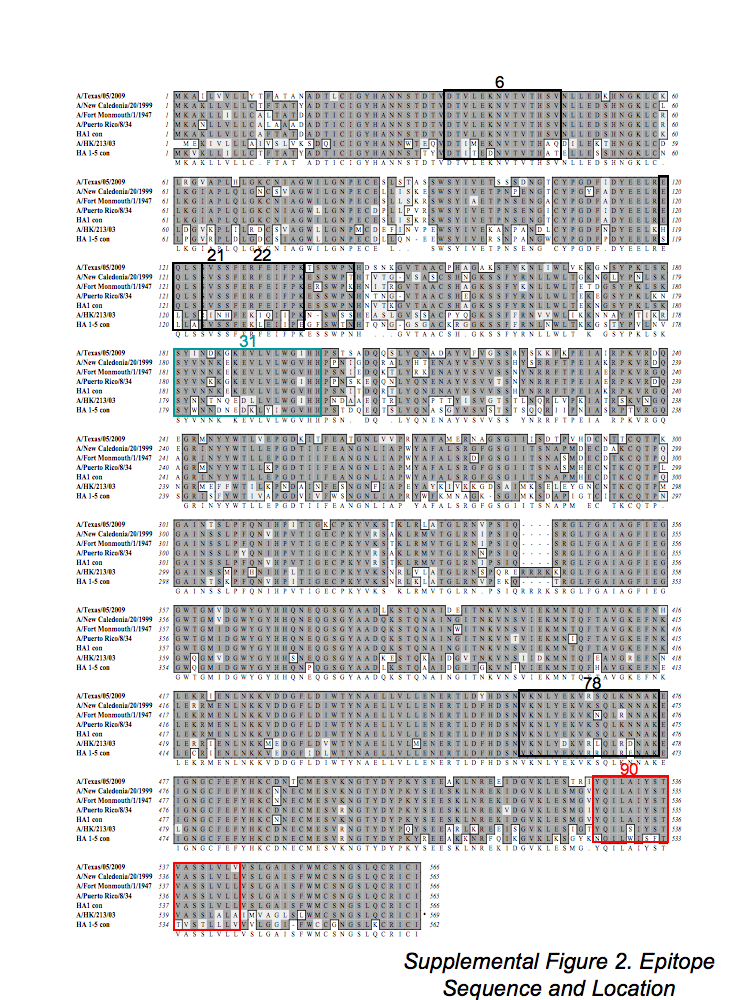

Supplement: Figure S2 — Alignment of the consensus influenza vaccine genes, the wildtype virus genes and the A/New Caledonia/20/99 HA proteins. Numbers represent the epitopes identified in Figure 4. Boxes represent the individual peptides that were recognized by immunized mice splenocytes. The green box represents a unique epitope recognized only by Ad-HA1-con immunized splenocytes and the red box represents the conserved immunodominant CTL epitope. Groups of 5 mice were used and error bars represent standard error. (TIF) [file pone.0018314.s002.tif]

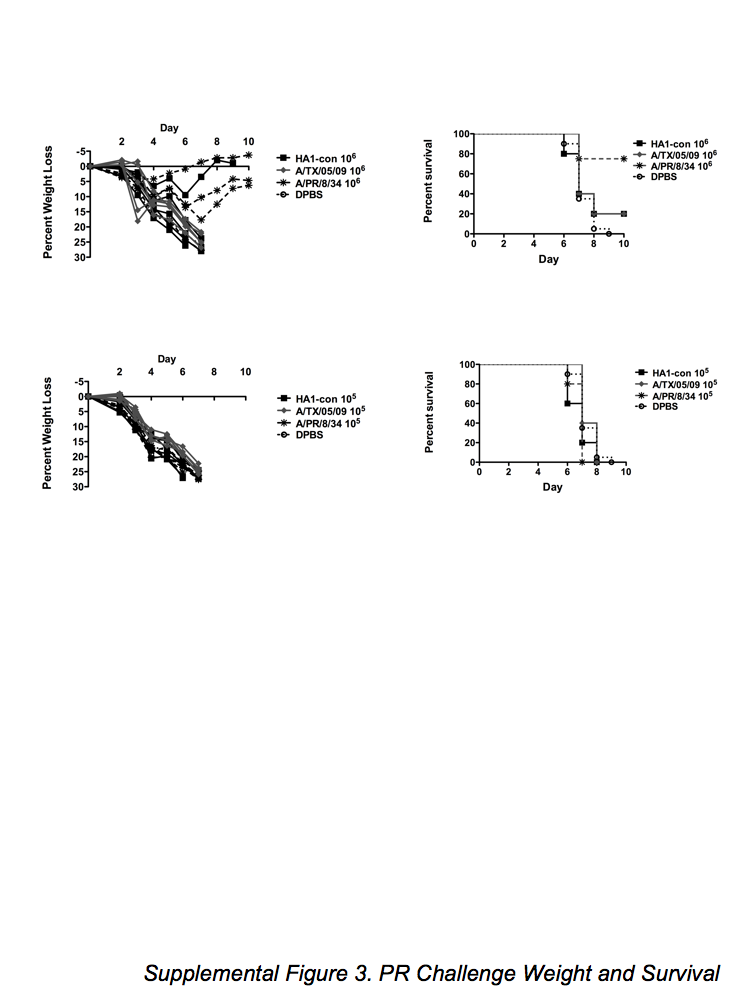

Supplement: Figure S3 — Dose-dependent prophylactic responses against a lethal A/PR/8/34 influenza virus challenge. Mice were immunized intramuscularly with various doses of A/PR/8/34, Ad-HA1-con, and A/TX/05/09 HA expressing virus. Three weeks after immunization the mice were challenged intranasally with 100 LD50 of influenza virus A/PR/8/34. Weight loss and death in mice immunized 106 vp are shown in A and B, respectively. Weight loss and death in mice immunized 105 vp are shown in C and D, respectively. The mean and standard error of the control DPBS immunized mice are shown. Mice exhibiting profound signs of disease and less than 75% of baseline weights were humanely sacrificed. (TIF) [file pone.0018314.s003.tif]

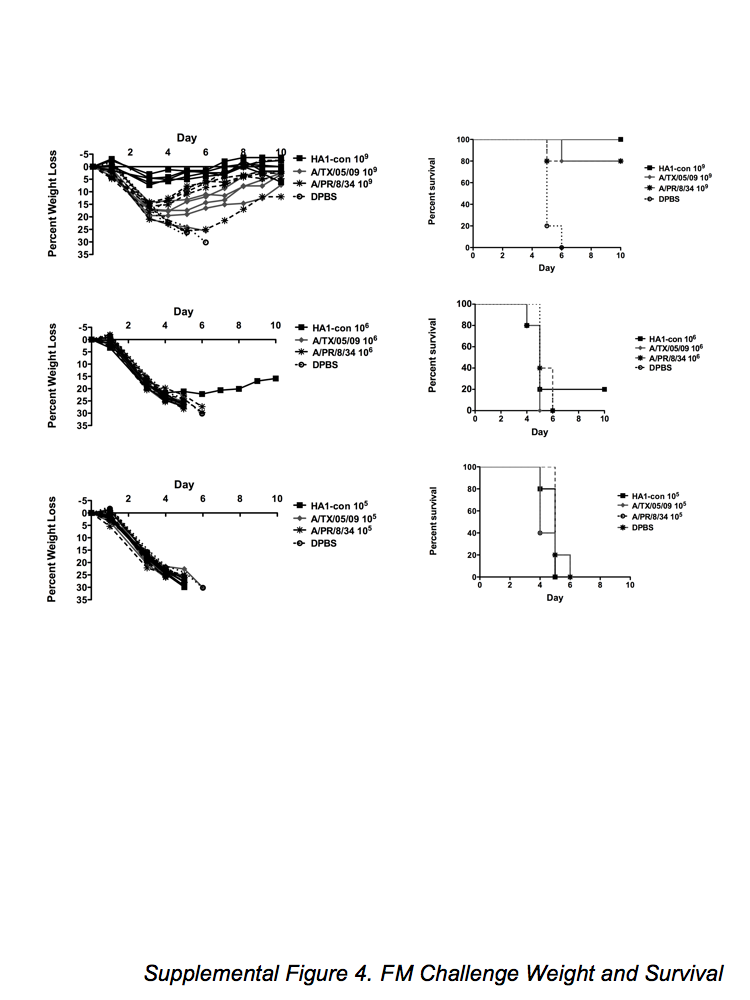

Supplement: Figure S4 — Dose-dependent prophylactic responses against a lethal A/FM/1/47 influenza virus challenge. Mice were immunized intramuscularly with various doses of A/PR/8/34, Ad-HA1-con, and A/TX/05/09 HA expressing virus. Three weeks after immunization the mice were challenged intranasally with 100 LD50 of influenza virus A/FM/1/47. Weight loss and death in mice immunized 10 vp are shown in A and B, respectively. Weight loss and death in mice immunized 106 vp are shown in C and D, respectively. Weight loss and death in mice immunized 105 vp are shown in E and F, respectively. The mean and standard error of the control DPBS immunized mice are shown. Mice exhibiting profound signs of disease and less than 75% of baseline weights were humanely sacrificed. (TIF) [file pone.0018314.s004.tif]

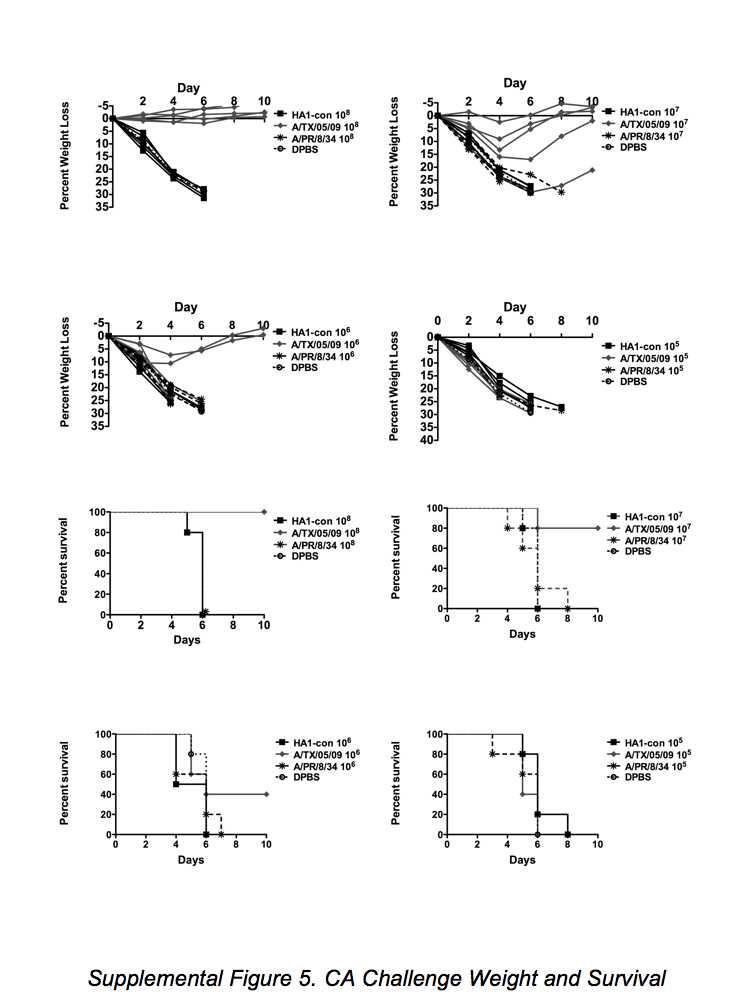

Supplement: Figure S5 — Dose-dependent prophylactic responses against disease after a lethal A/CA/04/09 Swine flu influenza virus challenge. Mice were immunized intramuscularly with various doses of A/PR/8/34, Ad-HA1-con, and A/TX/05/09 HA expressing virus. Three weeks after immunization the mice were challenged intranasally with 100 LD50 of influenza virus 2009 Swine Flu. Weight loss and death in mice immunized 108 vp are shown in A and B, respectively. Weight loss and death in mice immunized 107 vp are shown in C and D, respectively. Weight loss and death in mice immunized 106 vp are shown in E and F, respectively. Weight loss and death in mice immunized 105 vp are shown in G and H, respectively. The mean and standard error of the control DPBS immunized mice are shown. Mice exhibiting profound signs of disease and less than 75% of baseline weights were humanely sacrificed. (TIF) [file pone.0018314.s005.tif]
